# Conifer wood assemblage dominated by Podocarpaceae, early Eocene of Laguna del Hunco, central Argentinean Patagonia

**DOI:** 10.3897/phytokeys.156.54175

**Published:** 2020-08-21

**Authors:** Roberto R. Pujana, Peter Wilf, Maria A. Gandolfo

**Affiliations:** 1 Museo Argentino de Ciencias Naturales, Ciudad de Buenos Aires 1405, Argentina Museo Argentino de Ciencias Naturales Buenos Aires Argentina; 2 Department of Geosciences and Earth and Environmental Systems Institute, Pennsylvania State University, University Park PA 16802, USA Pennsylvania State University University Park United States of America; 3 LH Bailey Hortorium, Plant Biology Section, School of Integrative Plant Science, Cornell University, Ithaca, NY 14850, USA Cornell University Ithaca United States of America

**Keywords:** fossil forests, Huitrera Formation, Paleogene, Podocarpaceae, South America, wood anatomy

## Abstract

During the early Eocene, Patagonia had highly diverse floras that are primarily known from compression and pollen fossils. Fossil wood studies from this epoch are scarce in the region and largely absent from the Laguna del Hunco flora, which has a highly diverse and excellently preserved compression assemblage. A collection of 26 conifer woods from the Laguna del Hunco fossil-lake beds (early Eocene, ca. 52 Ma) from central-western Patagonia was studied, of which 12 could be identified to genus. The dominant species is *Phyllocladoxylon antarcticum*, which has affinity with early-diverging Podocarpaceae such as *Phyllocladus* and *Prumnnopitys*. A single specimen of *Protophyllocladoxylon francisiae* probably represents an extinct group of Podocarpaceae. In addition, two taxonomic units of cf. *Cupressinoxylon* with putative affinity to Podocarpaceae were found. Diverse Podocarpaceae taxa consistent with the affinities of these woods were previously reported from vegetative and reproductive macrofossils as well as pollen grains from the same source unit. Some of the woods have galleries filled with frass. Distinct growth ring boundaries indicate seasonality, inferred to represent seasonal light availability. Growth ring widths suggest that the woods came from mature trees, whereas the widths and types of some rings denote near-uniform temperature and water availability conditions.

## Introduction

The early Eocene Earth had warm climates worldwide (e.g., Zachos et al. 2001). In Patagonia, the southernmost region of South America, early Eocene ecosystems had highly diverse floras from mesothermal rainforest environments (Wilf et al. 2003, 2005, 2009; Barreda and Palazzesi 2007).

The volcanic-lacustrine strata of the Tufolitas Laguna del Hunco of the Eocene Huitrera Formation exposed at Laguna del Hunco in northwestern Chubut, central Patagonia, have long been known for their diverse and superbly preserved plant fossils (e.g., Berry 1925). According to previous paleobotanical studies, an extremely diverse mesothermal flora dominated by angiosperms, coupled with a significant presence of ferns and conifers, was present in the area (e.g., Wilf et al. 2003, 2005; Barreda et al. 2020). Among the conifers, Podocarpaceae (Wilf 2012, 2020; Wilf et al. 2017; Andruchow-Colombo et al. 2019), Cupressaceae (Wilf et al. 2009), and Araucariaceae (Wilf et al. 2014; Barreda et al. 2020; Rossetto-Harris et al. 2020) are well-represented in the flora. The modern biogeographic affinities of the Laguna del Hunco flora are diverse, and especially large concentrations of survivor genera are found in the tropical West Pacific region (e.g., Wilf et al. 2013).

Although fossil leaves, reproductive structures, and pollen are well documented, a comprehensive study of the woods from Laguna del Hunco is lacking. Petersen (1946) reported fossil trunks exposed in the upper portion of the fossil lake beds, but so far, the only described silicified specimen is a stem of the osmundaceous fern *Todea* from the southern exposures of the Tufolitas (Bippus et al. 2019; Bomfleur and Escapa 2019). In general, studies of Patagonian Eocene woods are scarce. From Argentinean Patagonia, Brea et al. (2009) described a conifer collected from the Koluel-Kaike Formation, and recently Pujana and Ruiz (2019) described an assemblage from the Río Turbio Formation with woods of Araucariaceae, several Podocarpaceae, Proteaceae, Cunoniaceae, and Nothofagaceae. From the Ligorio Márquez Formation in central-south Chilean Patagonia, Terada et al. (2006a) described a few woods of Araucariaceae, Podocarpaceae, and Cunoniaceae, and Terada et al. (2006b) described another small assemblage with Araucariaceae, Podocarpaceae, and Nothofagaceae from the Loreto Formation in southern Chilean Patagonia.

During a recent field season, we collected a large sample of fossil woods from the Tufolitas Laguna del Hunco, including both angiosperms and conifers. In this contribution, we treat the conifer woods from Laguna del Hunco for the first time. This work comprises the largest study to date of the Laguna del Hunco flora from permineralized wood specimens, otherwise known almost entirely from compression and palynomorph remains.

## Materials and methods

**Figure 1.**
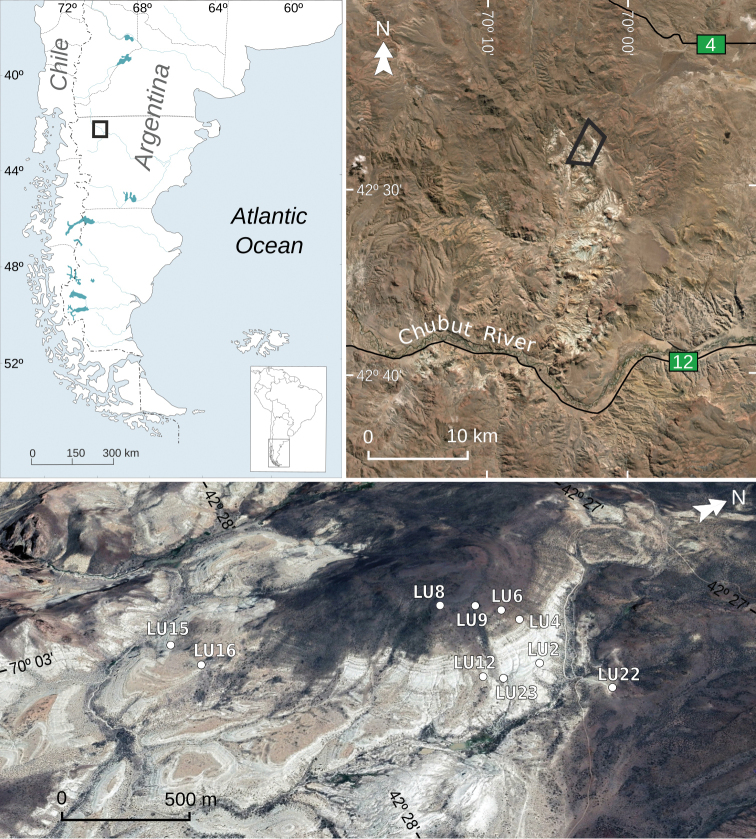
Location map and satellite images (Instituto Geográfico Nacional de la República Argentina, upper, and Google, CNES / Airbus, below) showing the Laguna del Hunco section and sampling locations. Scale in the satellite image below (tilted) varies across the map.

Fossil-wood samples were collected 3–5 December 2016 from 10 localities in the exposures of the Tufolitas Laguna del Hunco, Huitrera Formation, at Laguna del Hunco (Fig. 1; Table 1). The Tufolitas Laguna del Hunco are tuffaceous caldera-lake sediments that belong to the volcaniclastic-pyroclastic complex of the middle Chubut River (Aragón and Mazzoni 1997). The age of the Tufolitas Laguna del Hunco at Laguna del Hunco is constrained to the early Eocene (Ypresian) by the combination of a 52.22 ± 0.22 Ma ^40^Ar-^39^Ar age analyzed from sanidines in a tuff from the middle of the 170 m section, additional ^40^Ar-^39^Ar ages from the lake beds as well as associated units, and paleomagnetic data (Wilf et al. 2003, 2005; Wilf 2012; Gosses et al. 2020). The studied fossil woods were found on strata throughout the local Tufolitas section of Wilf et al. (2003; Fig. 1), including the upper third of the section that contains few compression fossils, and even the uppermost lake beds below the hill-capping Andesitas Huancache (per Aragón and Mazzoni 1997). Most specimens were found exposed on slopes, clean of attached sediment and with abraded surfaces, indicating that they were reworked downslope to an unknown extent from various possible source levels within the Tufolitas.

**Table 1.** Geographical coordinates of the localities where the fossils were collected.

Sampling locality	Geographical coordinates (DDM)	n° of conifer woods studied (identified to genus)
LU2	42°27.53'S, 70°02.26'W	1 (1)
LU4	42°27.51'S, 70°02.43'W	2 (1)
LU6	42°27.54'S; 70°02.48'W	4 (2)
LU8	42°27.68'S; 70°02.56'W	1 (1)
LU9	42°27.60'S; 70°02.52'W	2 (0)
LU12	42°27.67'S; 70°02.28'W	1 (0)
LU15	42°28.28'S; 70°02.92'W	1 (1)
LU16	42°28.23'S; 70°02.76'W	6 (1)
LU22	42°27.42'S; 70°02.09'W	7 (4)
LU23	42°27.63'S; 70°02.25'W	1 (1)

The 26 conifer fossil woods studied here (Table 2) are part of a larger collection of 87 wood samples. All studied specimens are decorticated and consist of permineralized (mostly by silica) secondary xylem, and their preservation is quite variable; only 12 of the 26 specimens could be assigned to generic level. Of the remaining specimens, 56 are identified as angiosperms (under separate study) and five, due to very poor preservation, could not be determined to any taxonomic group.

All specimens are housed at the Museo Paleontológico Egidio Feruglio, Trelew, Chubut Province, Argentina, repository acronym MPEF-Pb (Table 2). Thin sections of each sample bear the specimen number followed by a lower case series letter. We prepared thin sections (transverse, TS; tangential longitudinal, TLS; radial longitudinal, RLS) following standard techniques and studied them using light microscopy. Small fragments (radial views) of the samples were gold-coated and observed with scanning electron microscopy (SEM, Philips XL30 located in the Museo Argentino de Ciencias Naturales, Buenos Aires, Argentina). Microscopic images were taken with a Leica DM500 microscope with a Leica DFC420 camera. Images were processed with GIMP 2.8.22 software, and measurements from the images were taken with IMAGEJ 1.52 software.

**Table 2.** Wood anatomy of studied conifer samples. Locality (LU); Seriation index (Si); Contiguity percentage (Cp) [%]; Mean vertical diameter of radial pits (VDRP) [μm]; Mean tracheid tangential diameter (TTD) [μm]; Mean pits per cross-field (PxCF); Mean vertical diameter of cross-field pits (VDCP) [μm]; Mean ray height (RH) [cells]; Mean rays per mm (R×M). * indicates fewer than 15 measurements.

MPEF-Pb	Taxonomic unit	LU	Si	Cp	VDRP	TTD	PxCF	VDCP	RH	RxM
10694	*Protophyllocladoxylon francisiae*	2	1.25	88.1	19.2	44.5	1.97	14.8	5.6	3.5
10697	cf. *Phyllocladoxylon*	4	1.00*	5.6*	15.6	?	1.00*	11.4*	?	?
10700	Indeterminate conifer	4	1.00*	7.1*	14.6	?	?	?	?	?
10707	*Phyllocladoxylon antarcticum*	6	1.00	12.5	19.3	33.1	1.07	10.8	10.1	8.2
10710	*Phyllocladoxylon antarcticum*	6	1.03	8.5	20.9	29.5	1.11	12.0	7.9	8.4
10724	Indeterminate conifer	6	?	?	?	?	?	?	?	?
10725	Indeterminate conifer	6	?	?	?	?	?	?	?	?
10733	cf. *Cupressinoxylon* sp. 1	8	1.01	7.2	13.9	24.2	1.15	7.4	4.0	6.7
10736	Indeterminate conifer	9	?	?	?	?	?	?	?	?
10739	Indeterminate conifer	9	?	?	?	?	?	?	?	?
10742	Indeterminate conifer	12	?	?	?	?	?	?	?	?
10747	*Phyllocladoxylon antarcticum*	15	1.00	9.6	17.5	32.1	1.03	14.0	5.9	5.7
10748	Indeterminate conifer	16	?	?	?	?	1.00*	?	?	?
10749	cf. *Phyllocladoxylon*	16	1.00*	9.5*	16.8*	?	1.00*	13.7*	9.5	9.6*
10750	Indeterminate conifer	16	1.00*	12.5*	?	?	?	?	?	?
10751	Indeterminate conifer	16	?	?	?	?	1,00*	?	?	?
10753	Indeterminate conifer	16	?	?	?	?	?	?	?	?
10754	Indeterminate conifer	16	?	?	?	30.7	?	?	5.8	5.8*
10765	*Phyllocladoxylon antarcticum*	22	1.00	13.4	16.4	31.7	1.00	13.6	5.6	4.0
10766	Indeterminate conifer	22	?	?	?	?	?	?	?	?
10767	*Phyllocladoxylon antarcticum*	22	1.17	23.1	19.1	42.1	1.00	14.6	11.6	5.1
10771	Indeterminate conifer	22	?	?	?	?	?	?	?	?
10773	*Phyllocladoxylon antarcticum*	22	1.00*	5.3*	17.1*	31.2	1.00	12.9	6.2	8.1*
10775	Indeterminate conifer	22	1.00*	16.3*	15.1	?	1.00*	?	?	?
10776	*Phyllocladoxylon antarcticum*	22	1.00	10.6	17.5	32.5	1.06	13.2	9.9	6.3
10778	cf. *Cupressinoxylon* sp. 2	23	1.06	6.3	18.4	32.7	1.09	11.7	5.9	6.6

We followed the terminology of the IAWA Softwood Committee (2004) and the Si and Cp indices of Pujana et al. (2016) for describing wood anatomy. These two indices quantify the intertracheary pitting; e.g., Si = 1. 00 indicates that all the intertracheary pits are uniseriate, S > 1. 00 indicates that there are two- or more-seriate pits, Cp = 0% that pits are non-contiguous, and Cp = 100% that all pits are contiguous (Pujana et al. 2016). We also followed the definitions of Philippe and Bamford (2008) for classifying intertracheary pitting into the categories abietinean, mixed, and araucarian. In abietinean intertracheary pitting, most (ca. > 90%) of the pits in the radial walls are non-contiguous, are rounded in outline, and when in rows are opposite. In araucarian pitting, most (ca. > 90%) of the pits are contiguous and commonly alternate and angular in outline. Mixed pitting is when the pitting arrangement is between araucarian and abietinean. When possible, at least 15 measurements or observations of each character were made per specimen. Measurements are expressed as the mean followed by the range and mean standard deviation (SD) in parentheses. Measurements reported from species with more than one specimen were taken from all samples. Taxonomic determination was implemented using the criteria of Philippe and Bamford (2008) for delimiting conifer fossil-genera, while Bengston (1988) was followed for open nomenclature names.

For growth ring classification, we followed the scheme of Creber and Chaloner (1984). Minimum estimated diameters (MED) of the trunks/stems were measured directly on the sample or roughly calculated based on the curvature of the growth rings; when they had virtually straight growth ring boundaries, a 50 cm diameter was assigned.

## Systematic Paleontology

### Genus *Protophyllocladoxylon* Kräusel

#### Protophyllocladoxylon francisiae

Taxon classificationPlantaePinalesPodocarpaceae

Pujana, Santillana & Marenssi

06199D57-C350-55ED-8417-736AABFE0EDD

Figure 2A–F

##### Studied material.

MPEF-Pb 10694.

##### Locality.

LU2 at Laguna del Hunco (Fig. 1, Table 1), Chubut Province, Argentina.

##### Stratigraphic provenance.

Tufolitas Laguna del Hunco, Huitrera Formation (Ypresian, early Eocene).

##### Description.

**Figure 2.**
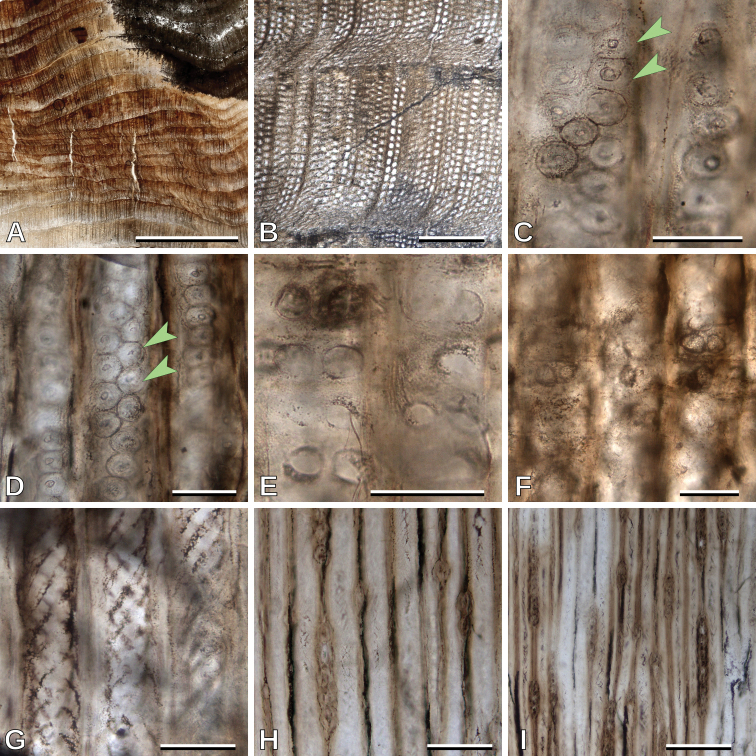
*Protophyllocladoxylon francisiae*, MPEF-Pb 10694: **A** Growth rings of type D (transverse section, TS) **B** detail of a growth ring of type D boundary (TS) **C** opposite (arrowheads) intertracheary radial pits (radial longitudinal section, RLS) **D** alternate (arrowheads) intertracheary radial pits (RLS) **E** and **F** cross-fields (RLS) **G** wall alteration of the secondary walls of tracheids (tangential longitudinal section, TLS) **H** uniseriate rays (TLS) **I** uniseriate rays (TLS). Scale bars: 5 mm (**A**); 500 μm (**B**); 50 μm (**C, D, E, F, G**); 100 μm (**H**); 200 μm (**I**).

Growth ring boundaries are distinct (Fig. 2A, B), latewood with 1–3 rows of tracheids (Fig. 2B). Tracheids are roundish to polygonal as seen in transverse section (Fig. 2B). Intertracheary pitting in radial walls is mixed, uni- to biseriate, predominantly uniseriate (Si = 1.25), contiguous (Cp = 88.1%), and mostly alternate, rarely opposite, when biseriate (Fig. 2C, D). Intertracheary pits are hexagonal to rounded in outline; 19.2 (13.8–24.6, SD = 1.9) μm in vertical diameter (Fig. 2C, D). Tracheid tangential diameter is 44.5 (30.3–61.2, SD = 7.0) μm. Cross-fields have 1–4, mean 1.9, pits per cross-field (Fig. 2E, F). Cross-field pits are circular with simple borders (rarely with narrow borders); 14.8 (11.8–18.4, SD = 1.8) μm in vertical diameter (Figs 2E, F, 6A). Horizontal walls of ray parenchyma cells are smooth (Fig. 2E). Wall alteration (not helical thickening) of the secondary walls of tracheids is observed (Fig. 2G). Average ray height is medium, 5.6 (1–13, SD = 3.2) cells high, rays are exclusively uniseriate (Fig. 2H, I) and with a frequency of 3.5 (2–5, SD = 0.9) rays per mm.

##### Remarks.

This specimen is characterized by its distinct growth ring boundaries, uni- to biseriate mixed intertracheary radial pitting, cross-fields usually with one or two mostly simple pits, relatively wide tracheids, uniseriate rays, and absence of resin-plugs and axial parenchyma. These characters indicate that this wood belongs to the fossil-genus *Protophyllocladoxylon*, because of the mixed radial pitting, simple large pits in the cross-fields, uniseriate rays, and smooth ray cell walls (Philippe and Bamford 2008). Conservation of the name *Protophyllocladoxylon* was recently proposed by Zijlstra and Philippe (2020). Among the more than 20 species of the genus, *P. francisiae* is distinguished by its distinct growth ring boundaries, uni- to biseriate and mixed radial pitting, and absence of axial parenchyma and resin plugs (Zhang et al. 2010; Pujana et al. 2014).

*Protophyllocladoxylon francisiae* was first described by Pujana et al. (2014) from material collected from the Eocene La Meseta Formation, Seymour/Marambio Island, Antarctica, and it was later reported from the Paleocene Cross Valley and Sobral formations that crop out on the same island (Pujana et al. 2015; Mirabelli et al. 2018). It is also present in the Eocene-Oligocene Río Turbio Formation, Santa Cruz Province, southern Patagonia (Pujana and Ruiz 2019). Interestingly, as is the case at Laguna del Hunco, this species is always a minor component of its floras and never dominates the assemblages.

The fossil-genus *Protophyllocladoxylon* is quite controversial. Vajda et al. (2016) suggested that *Protophyllocladoxylon* represents various unrelated botanical groups, principally because of its long temporal range from the Paleozoic to the Cenozoic (Zhang et al. 2010; see also Andruchow-Colombo et al. 2019). Pujana and Ruiz (2019) suggested that *P. francisiae*, in particular, could represent an extinct member of the Podocarpaceae because it has the general wood anatomy of the family but does not conform to any of the extant genera.

### Genus *Phyllocladoxylon* Gothan

#### Phyllocladoxylon antarcticum

Taxon classificationPlantaePinalesPodocarpaceae

Gothan

A43C9C00-E954-598D-AA48-937E2591DBDC

Figure 3A–L

##### Studied material.

MPEF-Pb 10707, 10710, 10747, 10765, 10767, 10773 and 10776.

##### Localities.

LU6, LU15 and LU22 at Laguna del Hunco (Fig. 1, Table 1), Chubut Province, Argentina.

##### Stratigraphic provenance.

Tufolitas Laguna del Hunco, Huitrera Formation (Ypresian, early Eocene).

##### Description.

**Figure 3.**
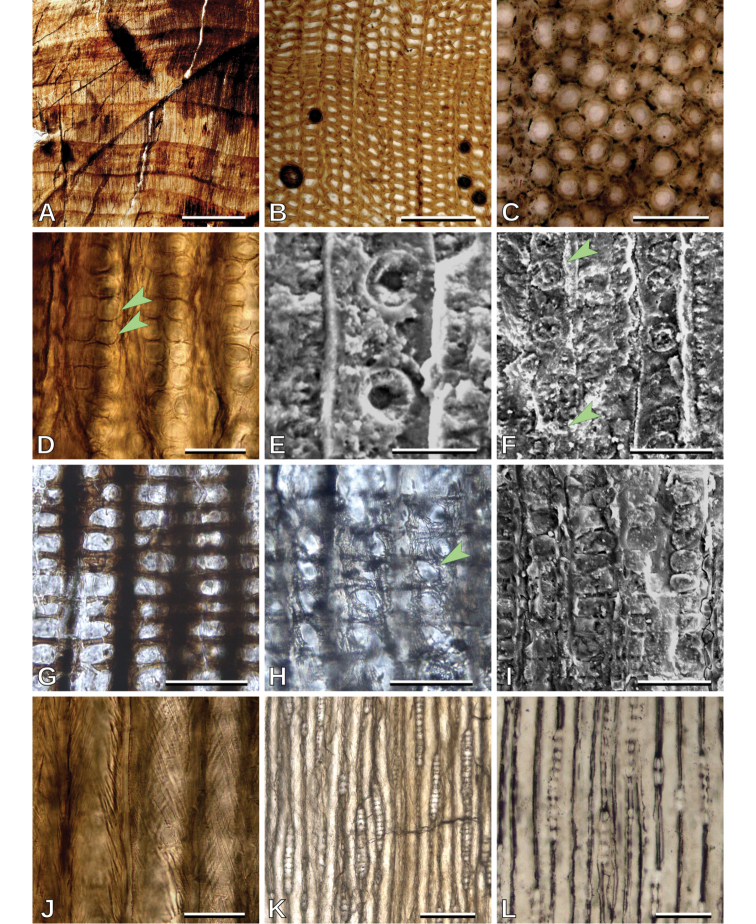
*Phyllocladoxylon antarcticum*: **A** Growth rings of type D (TS), MPEF-Pb 10747 **B** detail of a growth ring of type D boundary (TS), MPEF-Pb 10776 **C** detail of roundish tracheids (TS), MPEF-Pb 10765 **D** opposite contiguous biseriate intertracheary radial pits (arrowheads) (RLS), MPEF-Pb 10767 **E** uniseriate non contiguous intertracheary radial pits (scanning electron microscope, SEM), MPEF-Pb 10776 **F** uniseriate contiguous (arrowheads) and non contiguous intertracheary radial pits (SEM), MPEF-Pb 10776 **G** cross-fields with large simple pits (RLS), MPEF-Pb 10707 **H** cross-fields with large pointed and narrow-bordered pits (RLS), MPEF-Pb 10765 **I** cross-fields with large simple pits (SEM), MPEF-Pb 10710 **J** wall alteration of the secondary walls of tracheids (RLS), MPEF-Pb 10767 **K** uniseriate rays (TLS), MPEF-Pb 10767 **L** uniseriate rays (TLS), MPEF-Pb 10747. Scale bars: 5 mm (**A**); 200 μm (**B, K**); 100 μm (**C, L**); 50 μm (**D, F, G, H, I, J**); 20 μm (**E**).

Growth ring boundaries are distinct (Fig. 3A, B), latewood with ca. 3–10 rows of tracheids (Fig. 3B). Tracheids are roundish to polygonal as seen in transverse section (Fig. 3B, C). Intertracheary pitting in radial walls is abietinean, mostly uniseriate, rarely biseriate (Si= 1.03), mostly non contiguous (Cp= 11.9%), and opposite when biseriate (Fig. 3D–F). Intertracheary pits are rounded in outline; 18.3 (12.5–26.4, SD = 1.9) μm in vertical diameter (Fig. 3D–F). Tracheid tangential diameter is 33.2 (16.3–56.6, SD = 4.7) μm. Cross-fields have mostly 1, very rarely 2, mean 1.04, pits per cross-field (Fig. 3G–I). Cross-field pits are ellipsoidal with simple borders (rarely with narrow borders) and sometimes pointed; 13.0 (7.8–17.6, SD = 1.6) μm in vertical diameter (Fig. 3G–I, 6C). Wall alteration (not helical thickening) of the secondary walls of tracheids is observed (Fig. 3J). Horizontal walls of ray parenchyma cells are smooth (Fig. 3G, H). Average ray height is medium, 8.2 (1–34, SD = 5.0) cells high, rays are exclusively uniseriate (Fig. 3K, L) and with a frequency of 6.5 (3–11, SD = 0.2) rays per mm.

##### Remarks.

Abietinean intertracheary radial pitting and cross-fields with usually one large simple pit (Philippe and Bamford 2008) are the observed key characters, allowing confident placement of these Patagonian woods within *Phyllocladoxylon*. Distinct growth ring boundaries, absence of axial parenchyma and resin plugs, and predominantly uniseriate radial pitting are characteristics of the species *Phyllocladoxylon antarcticum* (Gothan 1908; Pujana et al. 2014).

Specimen MPEF-Pb 10767 frequently has biseriate opposite pits (Fig. 3D), and wider (in tangential section) tracheids, similar to *Protophyllocladoxylon*. However, most of its pits are non-contiguous (Cp= 23.1%), the growth rings are wider, and it has one pit per cross-field, all of which are features of the species *Phyllocladoxylon antarcticum*. Two other specimens, MPEF-Pb 10733 and 10778, are not very well preserved and are assigned to cf. *P. antarcticum* because two of the main characters (intertracheary radial pitting and cross-fields) are poorly preserved and therefore barely discernible (Table 2).

*Phyllocladoxylon antarcticum* is the most common species in our sample of conifer woods from Laguna del Hunco. In Patagonia, it was previously recorded in the Cretaceous (Nishida et al. 1990), Eocene (Pujana and Ruiz 2019), and in sediments of unknown ages (Kräusel 1924). On the Antarctic Peninsula, the fossil-species is the dominant component of the Eocene of Seymour/Marambio Island wood flora (Torres et al. 1994; Pujana et al. 2014) and a common component of wood floras from the Late Cretaceous of James Ross Island (Pujana et al. 2017), the Paleocene of Seymour/Marambio Island (Pujana et al. 2015; Mirabelli et al. 2018), and the Eocene of the Fildes Peninsula of King George/25 de Mayo Island (Torres and Lemoigne 1988; Oh et al. 2020).

Torres and Lemoigne (1988) suggested a possible relationship of *P. antarcticum* with the extant genera *Phyllocladus* Rich., *Dacrydium* Sol. ex G.Forst., *Microcachrys* Hook. *Prumnopitys* Phil., and *Podocarpus* Labill. Pujana et al. (2014) suggested affinities with several basal extant Podocarpaceae: the prumnopityoid clade (including *Phyllocladus* and *Prumnopitys*), *Microstrobos* Garden and LAS Johnson, and *Microcachrys* (Knopf et al. 2012); all of those taxa share with the fossils similar wood anatomy, abietinean radial pitting, and, predominantly, one large simple pit per cross-field (Pujana et al. 2014).

Recently, a compressed branch bearing phylloclades from Laguna del Hunco was assigned to the newly described fossil-genus *Huncocladus* Andruchow-Colombo et al., a stem relative of *Phyllocladus* (Andruchow-Colombo et al. 2019), and pollen having affinity with *Microcachrys* (Barreda et al. 2020) was also reported from Laguna del Hunco. These fossils could be related to *Phyllocladoxylon antarcticum*, although more evidence is necessary to confirm this hypothesis. *Prumnopitys andina* (Poepp. ex Endl.) de Laub., the only extant species of its genus from Patagonia, and *Phyllocladoxylon antarcticum* share similar wood anatomy (Pujana et al. 2017), and it is possible that the fossil-species could be related to the extant *P. andina*.

### Genus *Cupressinoxylon* Göppert

#### cf. Cupressinoxylon

Taxon classificationPlantaePinalesPodocarpaceae

sp. 1

982EFA68-5890-57F5-A09D-AA66F9C61E0C

Figure 4A–I

##### Studied material.

MPEF-Pb 10733.

##### Locality.

LU8 at Laguna del Hunco (Fig. 1, Table 1), Chubut Province, Argentina.

##### Stratigraphic provenance.

Tufolitas Laguna del Hunco, Huitrera Formation (Ypresian, early Eocene).

##### Description.

**Figure 4.**
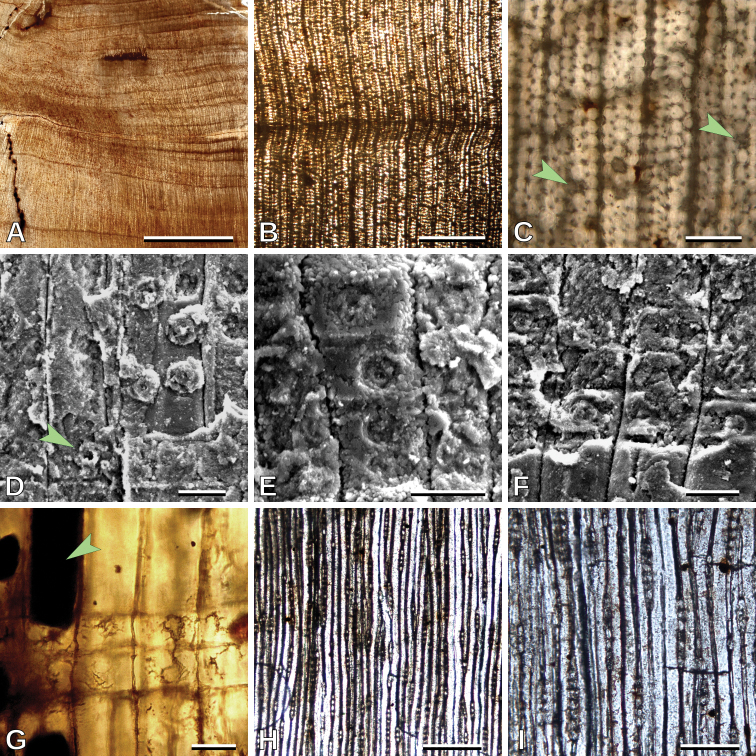
cf. *Cupressinoxylon* sp. 1, MPEF-Pb 10733: **A** Growth rings (TS) **B** detail of a growth ring boundary (TS) **C** Detail of roundish tracheids and axial parenchyma (arrowheads) (TS) **D** uniseriate non contiguous intertracheary radial pits and cross-field pit (arrowhead) (SEM) **E** and **F** cross-fields with bordered pits (SEM) **G** axial parenchyma (arrowhead) and cross-fields with bordered pits (RLS) **H** uniseriate rays (TLS) **I** uniseriate rays (TLS). Scale bars: 5 mm (**A**); 500 μm (**B**); 100 μm (**C, I**); 20 μm (**D, E, F, G**); 200 μm (**H**).

Growth ring boundaries are distinct (Fig. 4A, B), with a gradual transition from earlywood to latewood (Fig. 2B). Tracheids are roundish to polygonal as seen in transverse section (Fig. 4C). Intertracheary pitting in radial walls is abietinean, predominantly uniseriate (Si = 1.01), very rarely biseriate, non-contiguous (Cp = 7.2%), and opposite when biseriate (Fig. 4D). Intertracheary pits are rounded in outline; 13.9 (10.4–15.8, SD = 1.1) μm in vertical diameter (Fig. 4D). Tracheid tangential diameter is 24.2 (18.8–32.4, SD= 3.3) μm. Axial parenchyma is present, scarce, and apparently with a tendency to be tangentially zonate (Fig. 4C, G, I). Cross-fields have 1–2, mostly one, mean 1.2, pits per cross-field (Fig. 4D–G). Cross-field pits are circular and bordered, apparently the border is usually wider than the aperture, and the aperture is rounded; 7.4 (5.2–9.6, SD = 1.2) μm in vertical diameter (Figs 4D–G, 6B). Horizontal walls of ray parenchyma cells are smooth (Fig. 4E–G). Average ray height is medium, 4.0 (1–11, SD = 1.9) cells high, rays are exclusively uniseriate (Fig. 4H–I) and with a frequency of 6.7 (4–8, SD = 1.2) rays per mm.

##### Remarks.

Abietinean intertracheary radial pitting and apparently cupressoid pits in the cross-fields (bordered pits with the border wider than the aperture) indicate that this specimen belongs to the genus *Cupressinoxylon*, following Philippe and Bamford (2008). *Cupressinoxylon* includes wood with affinity to Cupressaceae and Podocarpaceae, with cupressoid cross-field pits (Pujana et al. 2014).

Cross-field pit border width is a key character of *Cupressinoxylon*. The poor preservation of this specimen prevents clear observation of the cross-field pits and also of the axial parenchyma walls; consequently, this specimen cannot be assigned with confidence to this fossil-genus and it is left as cf. *Cupressinoxylon*. Philippe and Bamford (2008) suggested that specimens in which the pit border is thinner than the aperture can also assigned to *Podocarpoxylon* Gothan.

The particular specimen studied here seems to be more similar to Podocarpaceae than to Cupressaceae because of the number of pits per cross-field. One, rarely two, pits per cross-field is common in the Podocarpaceae, whereas it is rarely observed in Cupressaceae (Tainter 1968; Greguss 1972; Meylan and Butterfield 1978; Roig 1992; Vidaurre Echeverría et al. 1987; Woltz et al. 1998; Vásquez Correa et al. 2010). Nevertheless, at this point we are not able to determine with certainty its affinity.

#### cf. Cupressinoxylon

Taxon classificationPlantaePinalesPodocarpaceae

sp. 2

CB16425D-DCC9-5A1F-B414-1004230C1463

Figure 5A–I

##### Studied material.

MPEF-Pb 10778.

##### Locality.

LU23 at Laguna del Hunco (Fig. 1, Table 1), Chubut Province, Argentina.

##### Stratigraphic provenance.

Tufolitas Laguna del Hunco, Huitrera Formation (Ypresian, early Eocene).

##### Description.

**Figure 5.**
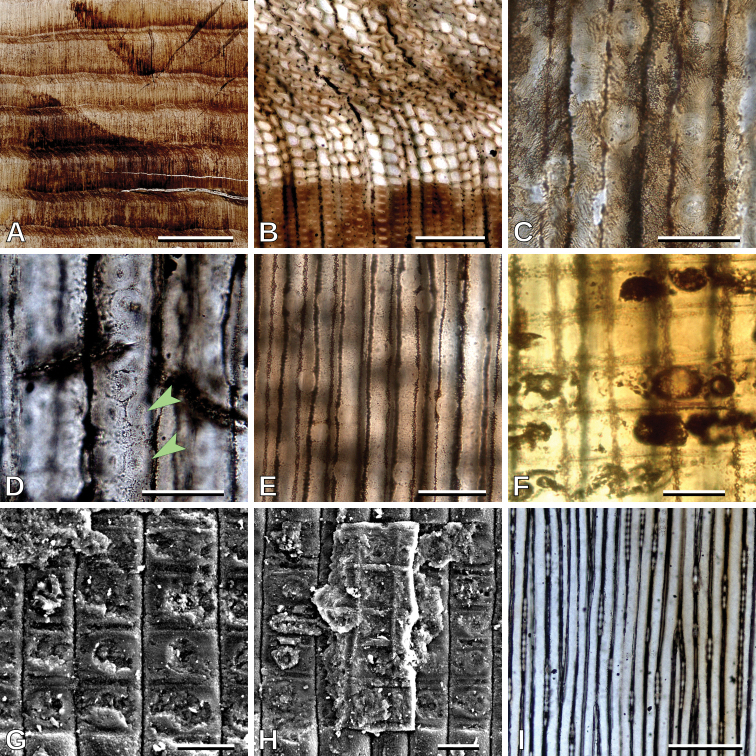
cf. *Cupressinoxylon* sp. 2, MPEF-Pb 10778: **A** Growth rings of type B (TS) **B** detail of a growth ring of type B boundary (TS) **C** uniseriate non contiguous intertracheary radial pits **D** uni- and biseriate intertracheary radial pits, opposite when biseriate (arrowheads) **E** uniseriate non contiguous intertracheary radial pits **F** cross-fields with bordered pits (RLS) **G** and **H** cross-fields with bordered pits (SEM) **I** uniseriate rays (TLS). Scale bars: 5 mm (**A**); 200 μm (**B, I**); 50 μm (**C, D, E**); 20 μm (**F, G, H**).

Growth ring boundaries are distinct (Fig. 5A, B), with a gradual transition from earlywood to latewood (Fig. 5B). Tracheids are mostly polygonal as seen in transverse section (Fig. 5B). Intertracheary pitting in radial walls is abietinean, uni- to biseriate, predominantly uniseriate (Si = 1.06), non-contiguous (Cp = 6.3%), and opposite when biseriate (Fig. 5C–E). Intertracheary pits are mostly rounded in outline; 18.4 (14.9–23.9, SD = 2.2) μm in vertical diameter (Fig. 5C–E). Tracheid tangential diameter is 32.7 (18.7–46.1, SD = 7.1) μm. Cross-fields have 1–2, mostly one, mean 1.1, pits per cross-field (Fig. 5F–H). Cross-field pits are circular and bordered, apparently the border is usually wider than the aperture, and the aperture is rounded; 11.7 (7.7–13.7, SD = 1.8) μm in vertical diameter (Fig. 4F–H, 6D). Horizontal walls of ray parenchyma cells are smooth (Fig. 4F–H). Average ray height is medium, 5.9 (2–15, SD= 3.1) cells high, rays are exclusively uniseriate (Fig. 5I) and with a frequency of 6.6 (4–9, SD = 1.4) rays per mm.

##### Remarks.

This specimen shares with cf. *Cupressinoxylon* sp. 1 the abietinean intertracheary radial pitting and bordered cross-field pits, and it also conforms to *Cupressinoxylon* according to Philippe and Bamford (2008). Unfortunately, it is also poorly preserved and could not be assigned with confidence to *Cupressinoxylon*. It differs from cf. *Cupressinoxylon* sp. 1 because sp. 2 has larger pits and lacks axial parenchyma. It also seems to be more similar to the Podocarpaceae than to Cupressaceae because they share the number of pits per cross-field (one, rarely two). In addition, in this specimen the mean diameter of the cross-field pits exceeds 10 μm, a feature present in South American species of *Prumnopitys* (Woltz et al. 1998; Vásquez Correa et al. 2010) and in other Podocarpaceae genera (Greguss 1955) but mostly absent in Cupressaceae, which usually have smaller pits (Greguss 1955, 1972; Roig 1992).

### Growth rings

Due to preservation, complete growth rings were only observed in a few specimens. Nonetheless, all the samples have distinct, well-defined growth ring boundaries (e.g., Figs 2A, 3A, 4A, 5A). Growth ring widths were measured where possible (Table 3). Growth rings are of types B and D (Table 3) of Creber and Chaloner (1984). The majority are type D, with abrupt transition from earlywood to latewood (Figs 2A, B, 3A), and only one specimen is type B, with a gradual transition from earlywood to latewood (Fig. 5A, B; Table 3). The mean ring width can reach 2.8 mm (Table 3). The minimum estimated age of the trees was grossly calculated to be 54 to 110 years, based on the curvature of the rings and the mean ring width (Table 3).

**Table 3.** Growth ring analysis of selected samples. Growth ring type (GRT); Mean width (MW) [μm]; Minimum and maximum growth ring width (Min.-Max.) [μm]; Standard deviation (SD) [μm]; Number of rings counted (n); Minimum estimated diameter (MED) [cm]; Minimum age (MA) [years], MA= (MED*10000/MW)/2.

MPEF-Pb	Taxonomic unit	GRT	MW	Min.–Max.	SD	n	MED	MA
10694	*Protophyllocladoxylon francisiae*	D	681	262–1314	327	21	15	110
10707	*Phyllocladoxylon antarcticum*	D	816	300–1324	307	19	12	73
10747	*Phyllocladoxylon antarcticum*	D	1400	371–3056	678	15	15	54
10778	cf. *Cupressinoxylon* sp. 2	B	2786	2258–3742	517	7	50	90

### Galleries

Two samples of indeterminate conifers (Fig. 7; Table 2) have frass-filled galleries inside. Specimen MPEF-Pb 10736 has a gallery ca. 1.2 mm in diameter, horizontally oriented and parallel to the growth rings, filled with apparently spherical, sometimes slightly ellipsoidal, coprolites of ca. 200–300 μm in diameter (Fig. 7A). Unfortunately, this sample is not well preserved, and the content may have been modified during the fossilization process. On the other hand, sample MPEF-Pb 10725 has a gallery of ca. 1.3 mm diameter that abruptly narrows and bifurcates (Fig. 7B), also horizontally oriented and parallel to the rays. The fill of this gallery has better preservation, and the content (frass) is clearly seen. The frass is compact, powdery, and made up of fragments of tracheids of ca. 100–300 μm in length, sometimes solitary and sometimes still united to adjacent tracheids (Fig. 7C).

**Figure 6.**
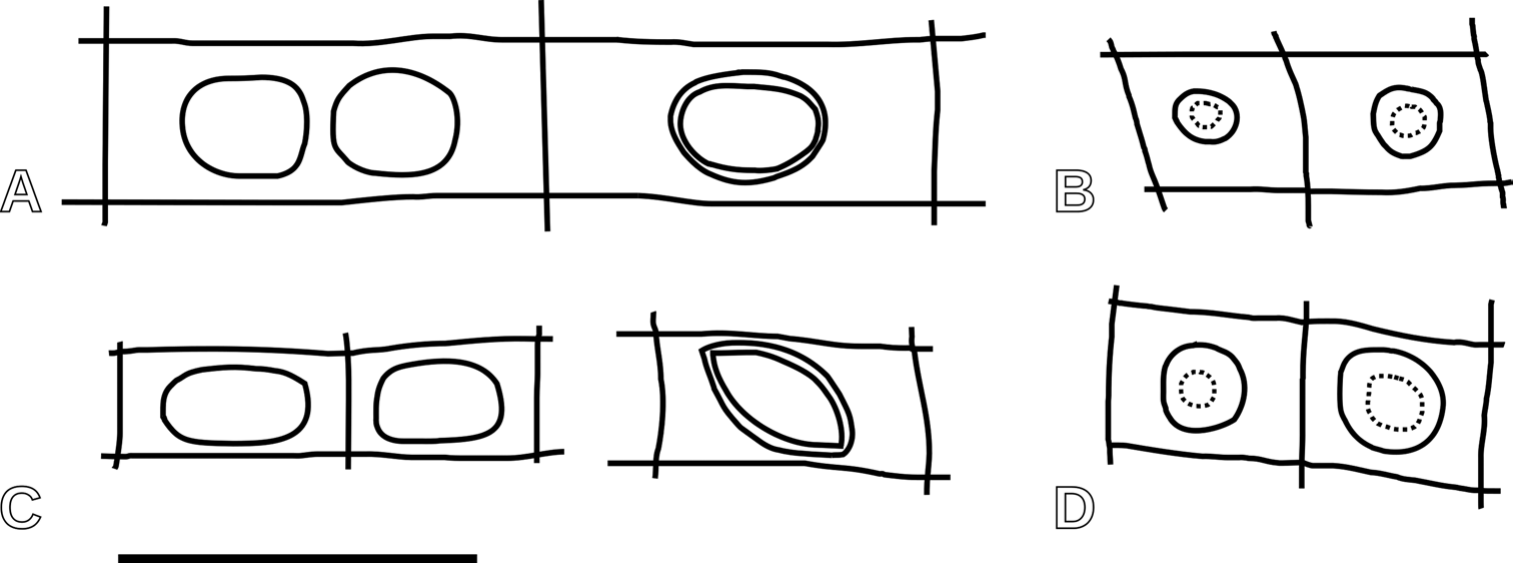
Schematic drawing of the cross-fields: **A***Protophyllocladoxylon francisiae***B** cf. *Cupressinoxylon* sp. 1 **C***Phyllocladoxylon antarcticum***D** cf. *Cupressinoxylon* sp. 2. Scale bar: 50 μm.

**Figure 7.**
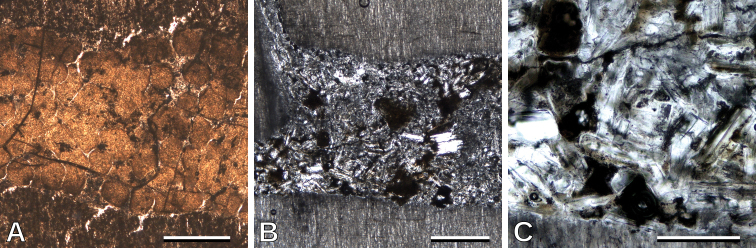
Galleries in two indeterminate conifer woods: **A** gallery filled with apparently spherical coprolites (TS), MPEF-Pb 10736 **B** gallery filled with compact frass (RLS), MPEF-Pb 10725 **C** detail of the frass (RLS), MPEF-Pb 10725. Scale bars: 500 μm (**A, B**); 200 μm (**C**).

## Discussion

The conifers represent about 30% of the total collected wood assemblage, indicating an important presence of this clade within the forest canopy. Even though not all the conifer woods could be identified, two taxa belong undoubtedly to Podocarpaceae, *Protophyllocladoxylon francisiae* and *Phyllocladoxylon antarcticum*, while the other two, cf. *Cupressinoxylon* sp. 1 and cf. *Cupressinoxylon* sp. 2, are putative Podocarpaceae. Consequently, we found a significant species richness (four types of woods) from only twelve samples that could be identified to genus.

The family Podocarpaceae was apparently the dominant conifer component within the Laguna del Hunco wood flora, with the caveat of small sample size. The family is also abundant and strikingly diverse at generic level in the intensively collected compression flora (>7,000 specimens), including remains of *Acmopyle* Pilg., *Dacrycarpus* (Endl.) de Laub., *Podocarpus*, and *Retrophyllum* CN Page as well as the extinct phyllocladoid genus *Huncocladus* (Wilf 2012, 2020; Wilf et al. 2005, 2017; Andruchow-Colombo et al. 2019). Moreover, the pollen grains of the Podocarpaceae are the most abundant among all gymnosperms so far recorded at Laguna del Hunco; they are represented by seven species within five fossil-genera (*Gamerroites* Archangelsky, *Dacrycarpites* Cookson and Pike, *Lygistepollenites* Harris, *Microcachryidites* Cookson, and *Podocarpidites* Cookson; Barreda et al. 2020).

Nevertheless, Araucariaceae, which are not yet known in the wood flora, are the most abundant conifer compression fossils at Laguna del Hunco, where *Agathis* Salisb. (formerly “*Zamia*”) and *Araucaria* Juss. compressions are each more common than any podocarp genus (Wilf et al. 2005, 2014; Rossetto-Harris et al. 2020). *Papuacedrus prechilensis* (Berry) Wilf et al. (Cupressaceae) is also well-represented in the compression flora (Wilf et al. 2005, 2009) but does not correspond exactly to any of the wood fossils because the living genus usually has one to five pits per cross-field (Greguss 1972).

This discrepancy in the family proportional abundances between the woods and compression macrofossils could result from many factors, most likely including the number of fossils studied, local variations of the source flora in time and space, and well-known differences in the taphonomic pathways for wood vs. other plant parts (e.g., Behrensmeyer et al. 2000). For example, many wood specimens were found in the upper part of the section at Laguna del Hunco (Fig. 1), and thus they must be younger than and possibly represent a slightly different source composition from the great majority of the compression samples, which are much more abundant in the middle part of the section (e.g., Wilf et al. 2003). The upper part of the section is more tuffaceous than the middle, probably reflecting rapid volcanic fill during the late phases of the caldera lake and a more frequently disturbed paleoenvironment (e.g., Aragón and Mazzoni 1997). We were only able to identify with certainty 12 specimens, and it is probable that with a larger wood sample, the occurrence of other conifer families from the compression flora, such as Araucariaceae and Cupressaceae, could be confirmed in the future. Nevertheless, the dominant status of the Podocarpaceae in fossil wood assemblages is a pattern observed for Patagonian and Antarctic woods during the Eocene (Pujana et al. 2014; Pujana and Ruiz 2017, 2019), whereas Cupressaceae and Araucariaceae, if present, were usually uncommon in all the wood floras of this epoch.

Podocarpaceae are today distributed mainly in the Southern Hemisphere and Malesia and are almost entirely restricted to rainforest or wet montane environments (de Laubenfels 1969; Hill and Brodribb 1999; Brodribb 2011; Cernusak et al. 2011). The family is ancient, with potential fossils from the Middle Triassic of Antarctica (e.g., Axsmith et al. 1998), and its fossil record from the Mesozoic through most of the Cenozoic is restricted to Southern Hemisphere land masses (Hill and Brodribb 1999). Interestingly, Podocarpaceae are often the most abundant gymnosperm group in living angiosperm-dominated rainforests (Brodribb 2011), as is the case for the Laguna del Hunco wood flora.

Growth ring boundaries of all samples are usually distinct (although some boundaries are not very well-defined), which indicates seasonality. The growth rings (type D; e.g., Figs 2A, B, 3A) are associated with the retardation of cambial activity, while the presence of type B (e.g., Fig. 5A, B) indicates a long growing season with an adequate water supply (Creber and Chaloner 1984). However, some Podocarpaceae with type D growth rings have wood that is not significantly affected by environmental factors, and can only be used in analyses of ring widths (Creber and Chaloner 1984). These types (B and D) of growth rings are consistent with the light regime at the paleolatitude of the sediments (about 47°S) and with previous paleotemperature and paleoprecipitation estimates based on leaf physiognomy and inferred drought-intolerance of many of the conifer taxa. The Laguna del Hunco compression assemblage, especially the conifer fraction, indicates no significant rainfall seasonality and very mild temperature seasonality (Wilf et al. 2003, 2005, 2009; Wilf 2012; Merkhofer et al. 2015).

The estimated minimum ages based on growth ring widths suggests that the specimens were mature trees at the time of deposition. We infer that the remains of *Protophyllocladoxylon francisiae* came from a tree older than 100 years (Table 3). In one sample, the type of growth ring (type B according to Creber and Chaloner 1984) and width of the growth rings reveal a significant and uniform growth of more than 5 mm in diameter annually (mean ring width 2.8 mm, widest ring of 3.7 mm, Table 3) which is similar to those of Podocarpaceae growing in wet Patagonian forests today (e.g., Rozas et al. 2016). Interestingly, the plant would have grown more than the global mean ring width of ca. 1.1 mm of extant conifers (Falcon-Lang 2005).

Galleries found in the woods were apparently produced by arthropod borers. They are filled with coprolites and particulate frass. This type of fill is produced by many types of arthropods (e.g., Platypodidae beetles, Tarno et al. 2011). The frass found is insufficient for its identification, mainly because the literature on wood debris produced by modern arthropods is scarce (Nuorteva & Kinnunen 2008) and mostly focused on northern hemisphere taxa (e.g., Hay 1968; Solomon 1977; Tarno et al. 2011). However, arthropod galleries are often found in Patagonian fossil woods (e.g., Genise 1995; Pujana et al. 2020), and mite coprolites were found in the permineralized *Todea* stem from southern exposures of the Tufolitas Laguna del Hunco (Bippus et al. 2019).

## Conclusions

Herein, we report the first taxonomic study of conifer fossil woods from the highly fossiliferous Laguna del Hunco exposures. The proportion of conifers in this fossil wood assemblage (ca. 30%) indicates a significant presence of this group within the paleoflora. We document the family Podocarpaceae with confidence as the dominant component of the Laguna del Hunco wood paleoflora. The family is represented by two fossil species, *Protophyllocladoxylon francisiae* and *Phyllocladoxylon antarcticum*. Additionally, two species assigned to the genus *Cupressinoxylon* (cf. *Cupressinoxylon* sp.1 and sp.2) are probably representatives of the family as well. Although sample size is small compared with the compression flora, these data strongly indicate that Podocarpaceae were important components of the Laguna del Hunco flora.

Podocarpaceae dominance in the fossil woods is consistent with diverse, abundant podocarpaceous macrofossil compressions and pollen grains previously described from the same section, including vegetative and reproductive structures related to several extant podocarp genera. However, in the Laguna del Hunco compressions, Araucariaceae are the dominant conifers, Cupressaceae are also well represented, and both families are found in the palynoflora. Neither family is yet known from the wood flora, presumably a result of lower sample size available for the woods by two orders of magnitude (10s of wood fossils vs. 1000s of compression fossils) or unknown taphonomic factors.

Growth rings indicate seasonality, probably because of the seasonal light regime at paleolatitude ca. 47°S, and mature tree development. The galleries found in two woods indicate arthropod interactions.

## Supplementary Material

XML Treatment for Protophyllocladoxylon francisiae

XML Treatment for Phyllocladoxylon antarcticum

XML Treatment for Cupressinoxylon

XML Treatment for Cupressinoxylon
